# Mobile-Enhanced Prevention Support Study for Men Who Have Sex With Men and Transgender Women Leaving Jail: Protocol for a Randomized Controlled Trial

**DOI:** 10.2196/18106

**Published:** 2020-09-22

**Authors:** Gabriel G Edwards, Cathy J Reback, William E Cunningham, Charles L Hilliard, Charles McWells, Sukrit Mukherjee, Robert E Weiss, Nina T Harawa

**Affiliations:** 1 Department of General Internal Medicine and Health Services Research David Geffen School of Medicine University of California, Los Angeles Los Angeles, CA United States; 2 Center for HIV Identification, Prevention, and Treatment Services University of California, Los Angeles Los Angeles, CA United States; 3 Friends Research Institute Los Angeles, CA United States; 4 Los Angeles Centers for Alcohol and Drug Abuse Los Angeles, CA United States; 5 Department of Preventative and Social Medicine Charles R Drew University Los Angeles, CA United States; 6 Center for Biomedical Informatics Charles R Drew University Los Angeles, CA United States; 7 Department of Biostatistics Fielding School of Public Health University of California, Los Angeles Los Angeles, CA United States; 8 Department of Psychiatry Charles R Drew University Los Angeles, CA United States

**Keywords:** HIV, MSM, transgender women, peer navigation, jail, substance use disorder, eHealth, PrEP, sexually transmitted infections, hepatitis C, mobile phone, smartphone

## Abstract

**Background:**

Men who have sex with men (MSM) and transgender women, particularly those who have experienced criminal justice involvement, have particularly high HIV burdens, and a majority of those in jail have substance use disorders (SUDs). MSM and transgender women also experience elevated rates of incarceration. Once community re-entry occurs, individuals are in a critical period for addressing potential risks of HIV and sexually transmitted infection (STI) acquisition and negative sequelae of substance use. Further, the impact experienced by one’s social and sexual networks experienced at the time of detention and release have important health implications for MSM and transgender women.

**Objective:**

The purpose of this study is to test a new intervention—Mobile-Enhanced Prevention Support (MEPS)—that involves a GPS-based mobile app called GeoPassport (referred to as GeoPass in practice), incentives, and peer support for promoting HIV prevention, substance use treatment, and use of related services.

**Methods:**

A two-arm, unblinded, randomized controlled trial will seek to enroll 300 HIV-negative MSM and transgender women, aged 18-49 years, with SUDs, who are either in jail or have recently left jail. Participants will be enrolled by study staff and randomized to the MEPS intervention group or usual care group. The intervention group will receive customized wellness goals in addition to GeoPass, cash incentives, and the support of a trained peer mentor for 6 months. Data collection will consist of a baseline survey and three follow-up surveys at 3, 6, and 9 months postenrollment, either in person or by phone or videoconference when necessary. The primary outcomes include establishing a primary care provider; being prescribed and adhering to pre-exposure prophylaxis (PrEP) for HIV; screening for HIV, STIs, and hepatitis C virus; and engagement in recommended treatment for SUDs. Secondary outcomes include obtaining treatment for any identified infections and avoiding recidivism.

**Results:**

Enrollment began in November 2019 and study completion is expected in 2023.

**Conclusions:**

This study will advance our knowledge base on patient navigation and peer mentor interventions. Peer navigation services have been studied for the treatment of HIV, but less often in the context of HIV and STI prevention among sexual and gender minority populations at the time of re-entry into the community from jail. The MEPS study will examine the acceptability and feasibility of combining peer mentor services with a mobile app to facilitate service utilization and participant–peer mentor communication. MEPS will assess patterns of PrEP uptake and utilization in MSM and transgender women leaving jail. The study will provide heretofore unavailable data from persons leaving jail regarding HIV PrEP, STI screening, substance abuse treatment, and service utilization patterns and experiences, including geocoded data for those in the intervention arm.

**Trial Registration:**

ClinicalTrials.gov (NCT04036396); https://www.clinicaltrials.gov/ct2/show/NCT04036396

**International Registered Report Identifier (IRRID):**

PRR1-10.2196/18106

## Introduction

In the United States, men who have sex with men (MSM) and transgender women face myriad and overlapping risks to their health and well-being. MSM and transgender women are disproportionately impacted by HIV, hepatitis C virus (HCV), and other sexually transmitted infections (STIs). In 2017, 70% of the new HIV diagnoses were among gay and bisexual men, and the majority of those individuals were non-White [1]. Transgender individuals face an HIV prevalence of 1.4%, nearly five times higher than that of the general population (0.3%), with the number going up to 19% when focusing on Black transgender women specifically [2]. MSM also face increased incidence of other STIs compared to men and women who have sex with women only [3]. A recent systematic review reported a high prevalence of other STIs among transgender women but noted that most of the studies were focused on sex workers and may not be representative of the larger population of transgender women [4]. MSM and transgender women populations generally are at increased risk of developing substance use disorders (SUDs) [5,6]. 

Those who have experienced criminal justice involvement have particularly high HIV burdens. MSM HIV prevalence estimates in jail populations are higher than in the general US population; in addition, MSM and transgender women populations experience elevated rates of incarceration [7,8]. Several factors contribute to the increased risk of HIV and incarceration among transgender women, including increased rates of survival sex, sex work, and experience of sexual violence [2]. Furthermore, a majority of people in jail have SUDs [9].

The period that begins following community re-entry from jail, or prison, has been associated with risky sexual and substance-using behaviors and is implicated in elevated rates of mortality in populations of people with criminal justice involvement. Among the starkest examples is a study documenting a nearly 13-fold increase in mortality risk in the first 2 weeks after release from a Washington State prison. Much of this excess mortality was attributable to overdose, with cocaine and opiates being the leading cause [10]. For MSM, recent incarceration may disrupt existing relationships and contribute to sexual concurrency [11]. As such, the re-entry period is critical for addressing potential risks of HIV and STI acquisition as well as negative sequelae of substance use. 

Although some demonstration projects have shown positive results in facilitating linkage to HIV care in the community for those leaving prison, there is a dearth of data about jails specifically [12-14]. Few, if any, randomized controlled trials (RCTs) have been published on effective interventions, particularly for substance-using people living with HIV leaving jail, and, by extension, those at risk for HIV, rather than those leaving long-term prison facilities. A recent RCT tested a peer-based intervention for HIV-positive individuals leaving jail that showed efficacy in preventing declines in viral suppression [15].

The provision of peer navigation services, including accompaniment, assistance with development of behavioral and self-care skills, and coaching clients to enhance patient communication with providers, has demonstrated promising results with HIV-positive patients [15-17]. However, we are not aware of any studies that focus on testing peer navigation services in sexual and gender minority populations with criminal justice involvement.

The purpose of this study is to inform an intervention designed to reach a high-risk population at a critical point for increased risk of HIV infection: MSM and transgender women who have SUDs and are leaving, or have recently left, jail. The study will test a new intervention called Mobile-Enhanced Prevention Support (MEPS) that involves a GPS-based mobile app called GeoPassport (referred to as *GeoPass* in practice), incentives, and peer support for promoting the use of HIV prevention and related services; the intervention will be compared to the case management services that these individuals can routinely access following jail release. It is hypothesized that the intervention will increase rates of service utilization; HIV, STI, and HCV screening; and use of pre-exposure prophylaxis (PrEP) for HIV prevention as compared to the standard-of-care case management, referred to below as the control group.

## Methods

### Study Aims

The study’s primary aims are as follows:

1. Measures of PrEP uptake:
  - Seek care with a primary care provider who can prescribe PrEP,
  - Obtain screening for PrEP,
  - Begin PrEP,
  - Adhere to PrEP, and
  - Remain on PrEP for at least 3 months.
2. Sufficient preventative screenings:
  - HIV screening every 3 months,
  - Bacterial STIs screening every 6 months, and
  - HCV screening at least once.
3. Enrollment in appropriate SUD treatment:
  - Received any SUD treatment postrelease,
  - Attended some SUD treatment services appropriate to their American Society of Addiction Medicine (ASAM) level, and
  - Met at least 70% of recommended treatment activities and frequencies for ASAM level of care.

The study’s secondary aims are as follows:

1. Obtain appropriate follow-up care for those who test positive for HIV, STI, and HCV.
2. Reduce recidivism during the study period.
3. Describe the temporal and geographic distribution of PrEP uptake and supportive social service utilization patterns of a postincarcerated jail population at high risk for HIV.

Study aims are measured at follow-up interviews that take place at 3, 6, and 9 months postenrollment.

### Study Population and Recruitment

This study will focus on the MSM and transgender women housed in the K6G (Keep Away Designation 6G) unit of the Los Angeles County (LAC) Men’s Central Jail and in residential recovery facilities in LAC.

The K6G unit houses individuals who self-identify as gay or bisexual men and transgender women. It is a voluntary unit in which people are housed for their own protection. Initial screening for entry occurs at jail intake. The unit was established based on a documented pattern of abuse of gay- and transgender-identified people by other inmates in the LAC jails. K6G is a protected custody unit, with limited access to other inmates. Individuals must pass additional screening regarding their sexual and/or gender identity to be housed in the unit [18]. The Los Angeles Centers for Alcohol and Drug Abuse (LA CADA) is funded via Medi-Cal (Medicaid in California) to provide SUD treatment services in the K6G unit. They operate the Substance Treatment And Re-entry Transition (START) program (ie, Project START), which was specifically tailored for these populations and includes both in-patient and transitional case management services.

**Figure 1:**
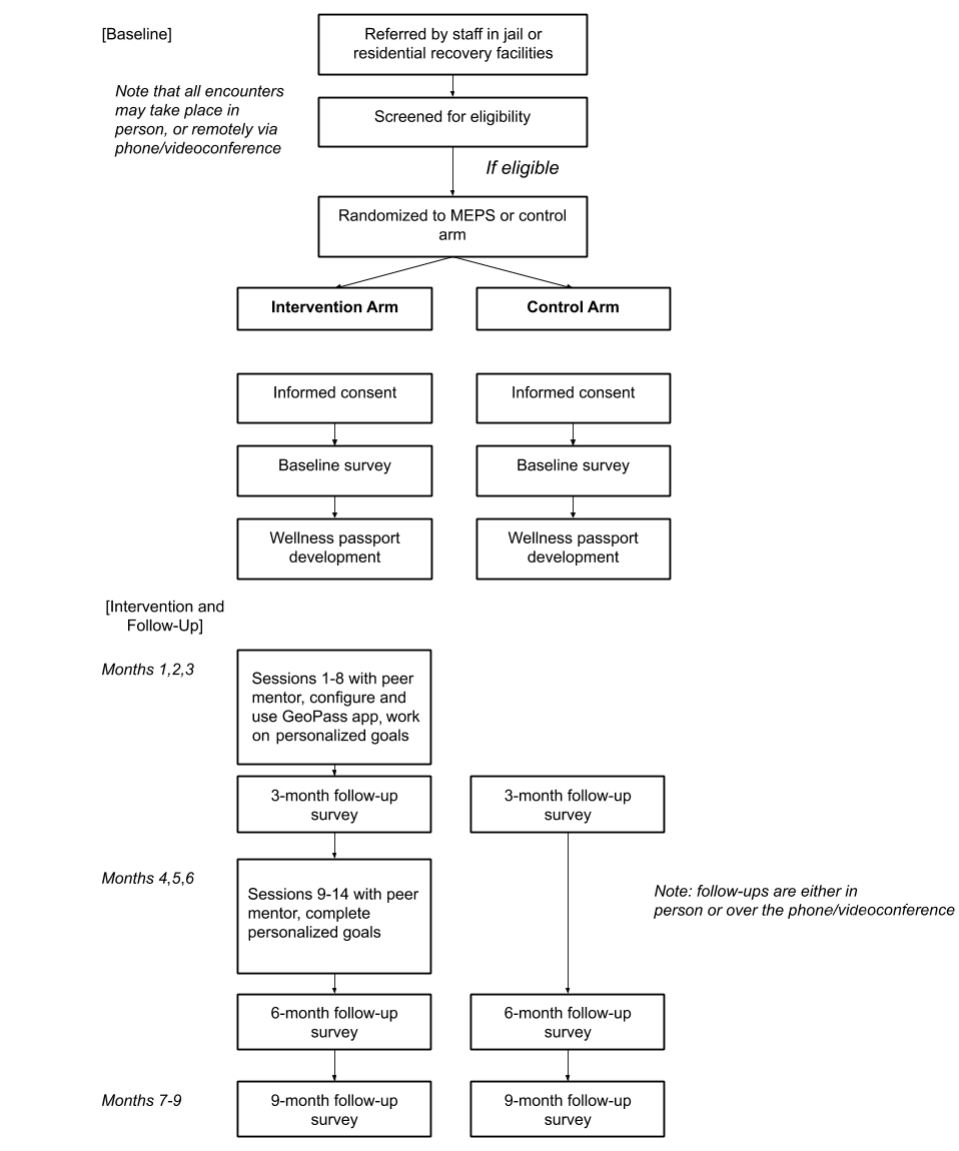
Mobile-Enhanced Prevention Support (MEPS) study flowchart.

The residential facilities are located throughout the Los Angeles metro area and serve as additional recruitment sites because they provide services to individuals who have recently been incarcerated. The community sites may provide residential treatment, recovery bridge housing, sober living, and/or supportive housing services. A flowchart of the MEPS study can be found in Figure 1.

We will enroll and randomize 300 individuals into the study who are recruited from jail or from residential facilities within 6 months postincarceration. We will conduct a trial comparing a control group (n=150) that is assigned to receive usual care to an intervention group (n=150) that receives GeoPass, incentives, and the support of a trained peer mentor for 6 months. GeoPass will provide participants with tools for tracking goals and progress toward meeting them, assistance in locating services, appointment and medication reminders, opportunities to provide feedback on service providers, and built-in tracking and distribution of rewards (ie, incentives) for service utilization. GeoPass will assist peer mentors in monitoring participants’ service utilization. The peer mentors will provide encouragement, role modeling, accompaniment to appointments, and assistance with goal setting, problem solving, and reducing logistical and psychosocial barriers to service engagement. Participants in both groups will be followed to assess whether those offered the MEPS intervention are more likely to utilize specific services.

### Study Eligibility

Study eligibility inclusion criteria are as follows: (1) housed in K6G unit or incarcerated in the previous 6 months and now residing in a residential facility with services (ie, residential treatment and recovery bridge housing) or supportive housing within 6 months of release, (2) aged 18-49 years, (3) screens positive for SUDs, (4) self-identifies as a man or transgender woman, (5) reports sexual intercourse with a male or a transgender woman in the 6 months prior to jail entry, (6) is likely to remain either in custody if in jail or at a residential recovery facility for at least 4 more days, but if in jail, then less than 3 more months based on scheduled court dates, current sentence, etc, (7) has not received an HIV diagnosis, based on self-report, and (8) plans to reside in LAC for the 12 months following enrollment.

Exclusion criteria are as follows: (1) does not have a smartphone and is not willing to obtain one postrelease, (2) is not able to speak and understand English, as the intervention is delivered in English, or (3) has insufficient reading skills to operate a mobile app.

For potential participants who do not have a smartphone at the time of enrollment, study staff will work with them to obtain a phone upon enrollment.

### Screening, Consent, and Enrollment Procedures

Study staff will communicate with residential facilities to identify potentially eligible participants. Staff at residential facilities, who possess the eligibility criteria, will assist in identifying eligible participants and talking to them about the study, using a brochure produced by the study to help introduce it to potential participants. Facility staff will fill out a referral form that includes a signature from the potential participant indicating their interest in being approached by the study staff. The study staff will arrange to meet the individual. Study staff (ie, a research associate or other member of the team) will meet with potential participants in a classroom area, an office, or another similar, semiprivate or fully private location within the jail or the residential facility. For enrollment by phone or videoconference, the interviewer will screen interested individuals and obtain consent from those who screened as eligible.

For in-person enrollment in jail, each participant will be called in individually and, where required, jail staff or a case manager will escort the participant from their dormitory to this space. Once inside the interview setting, for potential participants in jail, the jail staff will maintain some visual and very limited audio contact. They must maintain some audio contact in case of emergency; however, these personnel will not be able to hear the survey questions or the respondents’ answers. Los Angeles County Sheriff's Department (LASD) custody staff will only be told that it is a study to identify the best ways to keep people healthy following release.

The interviewer will introduce themselves and the study using the script in the in-person consent screen. If the individual expresses willingness to learn more after the brief introduction, they will finish the study description and continue with the screening questions. Randomization will coincide with informed consent, with participants learning the results at the same time as the interviewer. We will randomize participants to the two arms at a 1:1 ratio without any other restrictions. The data manager will pregenerate the random allocation sequence using a SAS program (SAS Institute Inc) designed for this purpose. Randomization allocations will be maintained in sequentially numbered, opaque sealed envelopes by the study director and interviewers for distribution as new study participants enroll. Randomization assignments will be opened and given sequentially after confirming eligibility and after consenting participants complete their baseline surveys. For participants recruited remotely, during enrollment, the data manager will send the allocation in an email attachment to be opened by the interviewer.

This double-consent approach has been used to examine the impact of residential substance abuse treatment length on both substance use and HIV risk behaviors [19]. This approach has been selected to minimize dissension and conflict among potential study participants assigned to the study arm that receives incentives and those assigned to the standard-of-care study arm.

After reviewing the consent form for the group to which the study participant was assigned and after providing potential participants with the purpose of the study, the study’s goals, potential risks, and safeguards for confidentiality, participants will be given the opportunity to ask questions and have them answered. Moreover, the interviewer will ask the participants questions to confirm their understanding by asking them to explain in their own words what the study entails, along with the study’s goals, main procedures, and risks and benefits, using an Evaluation to Sign a Consent Form for Research document produced by Charles R Drew University. Those able to respond satisfactorily will be asked to sign the informed consent form. Others may indicate a lack of interest at any time and return to their dormitories.

A locator form will be used to determine where and how study staff should attempt to reach participants once they leave jail or a residential facility in order to contact them for follow-up surveys and peer mentor meetings. In addition, participants will be encouraged to contact study staff via the study’s phone number or email following their release from custody. Release dates of those in jail will be solicited during the interview and tracked on publicly available inmate locator websites to establish target dates for the follow-up interviews, so the study team may reach them for follow-up once they are released from jail. On the locator form, participants will be asked to designate whether or not messages may be left and how they want study team members to refer to the study when leaving messages with their indicated contact information.

Participants will be asked to fill out and sign three release-of-information forms. One form is specific for LA CADA and allows the study team to access START assessments for participants who have gone through Project START. The second form is generic and allows us to contact organizations that the participant identified during the baseline interview. The third form allows future housing facilities to confirm the presence of the participant, should they move there during the study period.

### Baseline Assessment

The baseline surveys will be conducted by a trained research associate and may be performed in jail, in a residential facility in the community, or remotely by phone or videoconference as necessary. Participants will complete a 75-minute survey in a classroom, office, or other similar semiprivate or private location. The survey covers the following topics: (1) sociodemographics, (2) criminal justice history and status, (3) HIV and STI knowledge and risk perception, (4) psychosocial factors, such as medical mistrust and social support, (5) substance use, (6) self-efficacy, readiness, and motivation, (7) service utilization and needs, (8) sexual risk behaviors, and (9) health conditions. Most questions are asked during both the baseline and follow-up interviews; others are asked only during the baseline or the follow-up interviews.

In addition, we will collect basic information on participants’ current and prior arrests, including reasons for arrest, date of arrest, location of arrest, sentence length, release agency, and disposition codes, as well as some demographic information (ie, age, birth date, and race or ethnicity) from the publicly available LASD inmate locators; we will link this information with the survey data. By matching survey participants with these data, we will augment the surveys with record-based information on participants’ criminal justice involvement.

### Development of the Wellness Plan

The wellness plan will be based on an auto-generated assessment report from the baseline survey. This report will summarize potential sexual behavior and substance use risk factors; gaps in knowledge regarding HIV and STIs; history of PrEP use; prior testing for HIV, STIs, and HCV; lack of access to primary or mental health care; lack of social support; religious conflict; and unmet needs that the participant experienced prior to incarceration as well as health conditions for which they may need assistance in obtaining care postrelease.

Using the assessment from the baseline survey, the passport developer—either a trained full-time member of the study team or a trained substance abuse counselor from Project START or one of the residential facilities—will work with participants to determine which of the identified concerns they find most problematic and most motivates them to address postdeparture from jail or the residential facility. Goals related to PrEP uptake will vary based on each participant’s *stage of change* in this area and may range from accessing PrEP information from preidentified, online sites to reestablishing an existing PrEP prescription upon departure. Activities related to SUDs will depend on the recommended ASAM level of care based on results of assessments for SUDs as defined by the Diagnostic and Statistical Manual of Mental Disorders, Fifth Edition (DSM-5).

The 15-25-minute passport development process will result in a list of at least three specific goals and 8-12 activities that will facilitate reaching those goals, as well as the utilization of at least two of the health services that are part of the study outcomes. The process will also include education to ensure that the participant is aware of the importance of HIV, HCV, and STI testing and treatment; that they are informed about PrEP; and that any reported misconceptions about HIV and STIs that they may have are addressed. The study team will provide participants with a printed copy of each individual’s passport, augmented with detailed information on the referral agencies and resources discussed during the passport planning meeting. This passport will also be shared with the case managers of whatever program the participant is receiving residential services from.

The passport development process is rooted in both the transtheoretical model and motivational interviewing. It involves a client-centered approach to identifying participants’ needs, priorities, and readiness to change and starting the process of encouraging behavior change. Multimedia Appendix 1 shows a sample of the wellness passport.

### Control and Intervention Arms

#### Control Arm

Participants randomized into the control arm will continue to receive the usual care consistent with the setting they are in (ie, jail, residential treatment, supportive housing, etc). Participants recruited in jail will be enrolled in, or on the waitlist for, the LA CADA Jail Health Services program, called Project START, which is one of only four jail-based SUD treatment programs in LAC and is supervised and funded by the LAC Substance Abuse Prevention and Control Division of the Department of Public Health. Participants enrolled in the community will be housed in residential facilities that provide SUD services or supportive housing.

#### Mobile-Enhanced Prevention Support Intervention Arm

##### Overview of Arm

The MEPS intervention is designed to support, motivate, and facilitate engagement in preventive health care activities in the period of community re-entry after departing jail or a residential facility. The intervention involves three components, in addition to the standard of care: support from a selected peer mentor, incentives, and a newly developed mobile app. It is a client-driven approach, in which participants are encouraged to address the priorities and immediate needs that they identify through the app development process, especially social determinants of health that may discourage or undermine preventive health measures. A total of 14 peer mentor meetings are planned, either in person or remotely. Study staff will work with participants and any facilities in which they reside to ensure sufficient internet access to conduct peer sessions remotely, including obtaining a smartphone if needed.

The conceptual model for this peer mentor–led, mobile-enhanced intervention is based on an adaptation of Social Cognitive Theory (SCT) as applied to the ideal continua of care for HIV prevention and substance use. SCT is widely applied in HIV research because it helps explain how people acquire and maintain behavior change [20,21]. SCT underlying the peer mentor intervention holds that factors affecting the prevention continuum include the personal, behavioral, and environmental. Environmental factors include the experience of social support, social stigma, availability of care services, competing basic needs for care, and relationship with providers. The social environment also includes observational learning through peer mentor role models [22]. Personal factors include knowledge about HIV, STI, and HCV prevention as well as treatment for SUDs and the skills to perform and maintain related behaviors [23]. Behavioral factors include self-efficacy, outcome expectations, goal setting, and problem solving. We add the following to the classic SCT variables: barriers to, and facilitators of, HIV, STI, and HCV prevention and SUD treatment, such as homophobia, biphobia, and transphobia; spiritual conflict regarding sexuality; HIV stigma; and medical mistrust. These factors that been found in our previous studies and in the literature to be important predictors of uptake of PrEP; HIV, STI, and HCV screening; and SUD retention [24-28]. Participants assigned to the intervention arm will view photos of, and short introductions to, each of the available peer mentors; they will then select the peer mentor with whom they anticipate the greatest level of comfort and support based on the photo and introduction. They may also select a backup peer mentor, in case circumstances make a given one unavailable. The selected peer mentor will receive a copy of the participant’s passport and their contact information from the project director or research associate and will discuss with them the participant’s major issues and strengths.

Peer mentors will meet with their participants approximately every 1-2 weeks for the first 8 weeks to provide support and guidance and to work with them to address barriers in accessing services listed in their passport. Their initial focus will be in ensuring postrelease stability and linkage to services. They will also accompany them to key appointments, assist them in addressing other issues, and encourage productive communication with providers. Furthermore, the peer mentors will engage participants in ongoing evaluation of progress toward their goals and in considering new goals and passport modifications. Peer mentors will be trained to utilize motivational interviewing techniques to help participants resolve ambivalence that might undermine adherence to their services plan outlined in their passport.

In the third and fourth months of the intervention, peer mentors will interact with their participants less frequently than they had during the first 2 months. They will focus more on PrEP engagement and maintenance, HIV and STI screening, and addressing midterm and longer-term goals. During the final 2 months, the peer mentors will begin working with the participants to strategize for a successful transition from peer mentorship and incentives. This will include identifying other external motivators as well as internal motivators for engaging in prevention-related services and behaviors and, if appropriate, encouraging participants to actively identify and engage members of their social network as supports in ongoing maintenance of their health-related goals. Ensuring this smooth transition will be a key focus of the last month of engagement and will be emphasized at the final in-person meeting. All participants completing the final session and at least nine of their 14 scheduled sessions will receive printed certificates of completion.

##### GeoPass

GeoPass was developed by a MEPS coinvestigator (SM) who is affiliated with Charles R Drew University. It was designed from scratch using Java for Android devices, using Swift for iOS devices, and using ASP.NET for mobile web apps. The participating institutions are listed in the *About the App* section within GeoPass, including the logos of each organization. The app went into production in November 2019, at the start of recruitment; version 1.2 incorporated a geosensing feature update and version 1.3 incorporated geofencing feature additions for all providers.

Intervention participants will download GeoPass and receive their own log-in credentials. The app is downloadable from the Apple Store or Google Play free of charge and is only usable by study participants who are registered by the study team. In the first encounter with their peer mentor, participants will be oriented to the app and its features and encouraged to use it at least once per week. In general, the app is not meant as a stand-alone feature but a tool to facilitate the partnership between the participant and the peer mentor.

The app will create a mobile *passport* that incorporates personalized participant goals with other features that facilitate and motivate the accessing of needed services. These features include reminders, access details for service providers, positive automated feedback when services are utilized and goals attained, and messages from the peer mentors. GeoPass will require participants to provide feedback on services accessed in order to obtain the associated cash incentives. The cash will be transferred to the participant’s reloadable bank card. The feedback will involve closed-ended responses to four short questions and the opportunity to enter narrative feedback. Geolocation will validate service utilization using the smartphone’s built-in GPS capability and will ping participants to complete the feedback surveys when they attend providers or agencies that are part of an extensive database of local providers compiled for this purpose.

GeoPass will allow peer mentors to view the data for their participants so that they can tailor the subsequent guidance and support that they offer them. Peer mentors will receive push notifications and be able to follow up with their participants in real time via a dashboard that they can access via a peer mentor portal in the app. Geolocation data collection is triggered based on matches to the database of Los Angeles area service providers developed for the study. This database is built and maintained by study staff.

We intend to monitor the following indicators of app use:

1. Number of messages between each peer mentor and participant.
2. Number of surveys completed.
3. Percentage of surveys completed.
4. Number of goals added at baseline and over time.

The use of stored geolocation data will enable us to address secondary aim #3 by describing the temporal and geographic distribution of PrEP uptake and supportive social service utilization patterns of a postincarcerated jail population at high risk for HIV.

**Figure 2:**
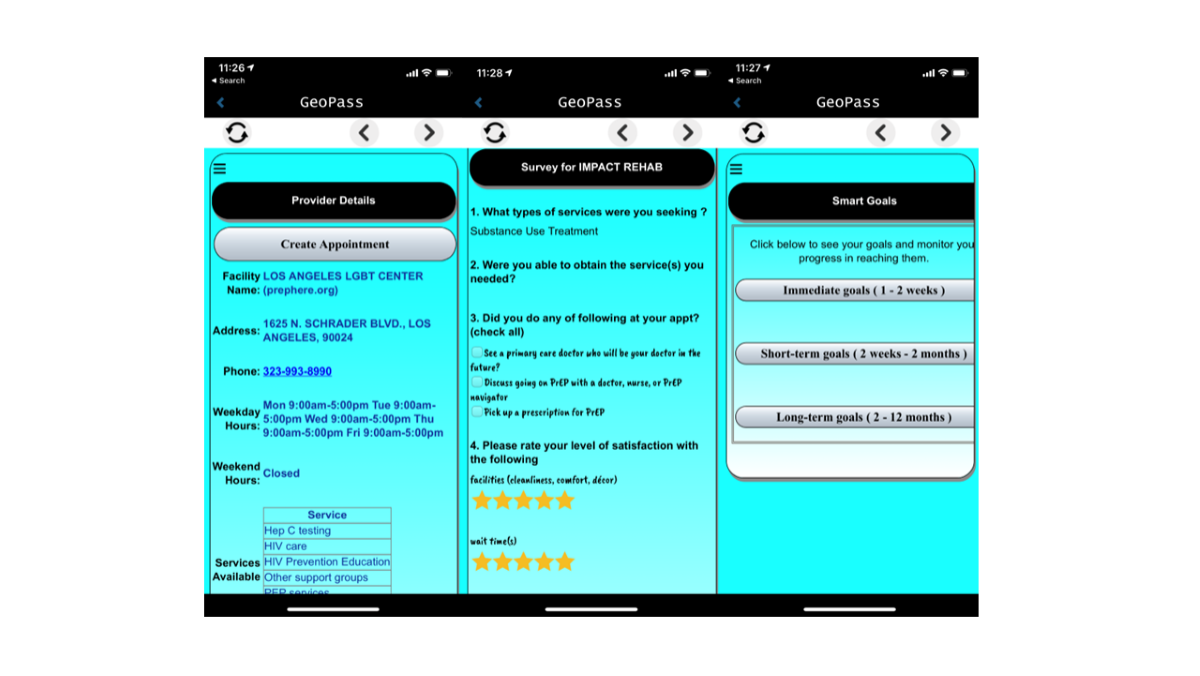
Screenshots of the GeoPass app.

Quality assurance for GeoPass incorporated multiple elements. A project consultant acted as a quality assurance specialist and wrote automated tests while the developer was writing code. Four different automated tests were used: (1) static code analysis using Lint and Sonar, (2) executing unit tests to validate whether each unit of the software performed as designed, (3) executing user interface integration tests to ensure that app components were correctly integrated, and (4) virtual device testing to find crashes in Android apps. Every piece of code written by one developer was approved by both Google Play and the Apple Store, and manual testing of the app was done based on specific use cases created by the project consultant acting as quality assurance specialist. Figure 2 displays screenshots taken from GeoPass as they appear on a smartphone.

##### Incentives

Over the course of the study, intervention arm participants may earn up to US $500 total in reloadable bank card incentives for completing passport activities. Most of these activities will take place over 6 months and are each valued at US $5-$15. These include medically related visits (ie, HIV, STI, and HCV screening and PrEP evaluation), substance use treatment appointments, in-person meetings with their assigned peer mentor or case manager, and nonmedically related items on their passports (eg, support group meetings for gay or bisexual individuals, MSM, or transgender women or training sessions at an employment service center). Certain categories of passport activities, such as substance abuse treatment services and *other*, are expressed as a range because they will vary from participant to participant; they will be determined individually in consultation with the peer mentor, participant, and the participant’s case manager, as applicable. A full schedule of different types of activities and the amount of accompanying incentives is provided in Table 1.

The passport’s incentive structure is designed to provide more compensation for those study components that are directly related to HIV and STI biomedical intervention or prevention; however, the largest portion of incentives is for SUD services that people with SUDs are recommended to access much more frequently. Frequency for HIV screening (every 3 months), STI screening (every 6 months), and HCV screening (at least once) is based on the Centers for Disease Control and Prevention recommendations for MSM at increased risk for HIV [26]. For the SUD-related services, participants will only be compensated for the number of visits recommended per their ASAM level of care. In addition, the incentives are intended to encourage initial linkage visits to other needed services and are then capped in order to avoid a situation in which participants repeatedly complete incentivized activities after they are no longer beneficial. Once participants take part in a particular service, resolve barriers to service access with a peer mentor, and identify providers they like, it is hoped that they will continue to engage with those services, as needed, regardless of the opportunity for an incentive.

**Table 1:** Incentive schedule.

Passport activity	Maximum number of times the activity can be completed	Amount, $
Link to a primary care provider who will prescribe PrEP^a^	1	15
PrEP screening and evaluation	1	15
Begin PrEP	1	15
Hepatitis C virus test	1	15
Sexually transmitted infection tests	2	15
HIV tests	3	15
Substance abuse treatment services, including AA^b^ and NA^c^ meetings, or meetings with case managers	8-14^d^	10
Meetings with peer mentors	14	5 or 15
Other (eg, visits to DPSS^e^, job training programs, and counseling)	6-10^d^	15

^a^PrEP: pre-exposure prophylaxis.

^b^AA: Alcoholics Anonymous.

^c^NA: Narcotics Anonymous.

^d^This is expressed as a range because activities will vary from participant to participant and because there is a cap on the total possible compensation.

^e^DPSS: Department of Public Social Services.

### Follow-Up Assessments

At 3, 6, and 9 months following the beginning of the intervention, participants will be asked to participate in follow-up interviews to examine changes in behaviors and rates of service utilization; snacks will be provided. Those who are released and then reincarcerated at the scheduled times for their follow-up interviews will be interviewed in custody in the same settings and using the same procedures as described for the baseline interviews that are conducted in custody.

### Compensation for Study-Related Activities

For participants in both arms who are in jail, a small stipend will be placed on the participants’ inmate commissary accounts (US $25), in compensation for their completion of the baseline survey and any follow-up surveys completed in custody due to reincarceration. Follow-up interviews conducted in the community will be compensated at the rate of US $50. Those participants who remain in jail for more than 3 months but less than 9 months will undergo a second, shortened version of the baseline survey and will receive an extra US $15 placed in their commissary accounts.

Participants interviewed outside of custody will receive US $25 cash for the baseline survey; they will receive US $50 cash compensation for each follow-up survey to account for the increased transportation and opportunity costs associated with participation postenrollment. Additional compensation will be provided throughout the study period for maintaining contact with the study team. Participants in both study arms will be given US $10 per month for initiating contact with the study team within 48 hours of departing jail or a residential facility and providing their up-to-date contact information during each subsequent month through month 9 of follow-up. All participants will receive a *Welcome Home Kit* containing personal items, such as condoms, a battery pack for their phone, and hygiene items of their choosing, worth approximately US $15 in retail value.

Enrolled participants may have an opportunity to refer other individuals for enrollment, for which they can receive bonuses of up to US $75 for 3 enrolled participants.

### Outcome and Statistical Data Analysis

#### Modeling Outcomes

Each outcome will be analyzed via intention-to-treat longitudinal analyses of all available data from the baseline and 3-, 6-, and 9-month follow-up interviews; the mobile app; and the abstracted record data on utilization, as appropriate. Weiss [29] describes our general approach to modeling longitudinal data. For PrEP cascade outcomes, the model is a logistic generalized linear mixed model (GLMM) with a random intercept for participants and a binary 0-1 outcome, with 1 indicating success (eg, screening for PrEP) and 0 indicating a lack of success (eg, not screening for PrEP). The first hypothesis is a test for each outcome of differences at 6 months; secondary hypotheses are tests of differences at 3 months and at 9 months. At baseline, groups have not been affected by interventions; thus, all groups will be the same. Subgroup analyses will be conducted to understand how the intervention outcomes differ by groups. A GLMM random intercept model can be fit in the SAS software PROC MIXED (SAS Institute Inc); if there are problems fitting the data in PROC MIXED, we will move to Bayesian software, such as JAGS in R (The R Foundation) [30].

To analyze visit counts, we will use a Poisson GLMM with log link and an offset equal to the log of the time covered by each observation, with a mean intensity (ie, mean count) of health care visits per unit time. The advantage of this over binary logit GLMM modeling, with 1 (*yes*) being equal to having had at least one visit in the past 3 months versus 0 (*no*) being equal to having no visits in the past 3 months, is that the binary model penalizes or rewards participants inappropriately for going a few days longer or shorter than 90 days in between visits; in addition, the logit model does not accommodate unbalanced follow-up times.

#### Modeling Issues and Sensitivity Analyses

Sensitivity analyses will include predictors that are predictive of missing visits or whose average levels differ across study arms. Predictors predictive of missingness are likely to occur; satisfactory randomization should prevent average predictor levels from differing across intervention groups. We will analyze missing visits as a binary outcome with a logistic random intercept regression model. Predictors will be baseline demographics and behavior variables. Examples of predictors are as follows: whether the participant had tested for HIV in the 3 months prior to baseline, age <30 years versus ≥30 years, type of SUD, ASAM Levels I and II versus Levels III and IV, stimulant user versus not, race and ethnicity, preferred substance, and education level.

Variables that are significant predictors of missing visits will be included as predictors in sensitivity analyses of intervention effects. If substantial missingness occurs in key baseline predictors, we will use multiple imputation to fill in the missing predictors. The missing visit analysis omits the baseline time point, as the baseline observation is required. To evaluate randomization, we analyze the key baseline predictors as outcomes in a one-way analysis of variance. If some of the predictors differ by intervention arm, they will be included in sensitivity analyses.

#### Power Calculations

We aim to enroll 300 total participants in the study, 150 in each group. With this sample size, and allowing for 20% dropout, we can detect a difference between the two groups of 18% (eg, 41% versus 59%) with .80 power at 2-sided α=.05. For rare events, such as initiation of PrEP, we can detect a difference of 14% between the control and intervention groups. We expect a fair amount of loss to follow-up, so we also calculated power for 25% attrition, leaving 112 participants per group.

#### Multiple Comparisons

We have listed 11 primary outcomes at 6 months for the three specific aims. To adjust for multiple comparisons, we propose to use a new methodology proposed by Harwood et al [31]. For 11 primary outcomes, we expect to reject, on average, 0.55 (ie, 11 × 0.05) null hypotheses, even in the case of no difference between arms. The Harwood et al methodology uses a correlated Bernoulli test of H_0_: *P*=.05 for 11 outcomes that reject the null hypothesis of no effect when 3 or more of the 11 tests are significant at *P*=.05. Rejecting this null hypothesis means that the interventions are not the same, gives a type I error of .05, and accommodates correlations across outcomes. We then declare individual outcomes that produced a *P* value below .05 as significantly different across arms as well. We will separately test outcomes at 3 months, 6 months, and 9 months using this methodology.

### Participant Safety

The principal investigator and the research team will make every effort to ensure the safety of participants and others throughout the duration of the study. A distress protocol was developed to guide study staff in situations where a participant indicates a desire or plan to harm himself or herself. In the community, peer mentors will also be equipped with emergency referrals to provide where necessary and possible, for resources such as emergency food, housing, domestic violence, and medical and psychiatric care.

### Ethics Committee Approval

This study was approved by the Institutional Review Board at the University of California, Los Angeles (UCLA), as well as the LAC Department of Health Services. The study has been registered with ClinicalTrials.gov (NCT04036396).

## Results

Recruitment began in November 2019 and will run through April 2022. Findings will be disseminated starting in January 2022 until March 2023, with the final report submitted in March 2023.

## Discussion

The MEPS study will address several important gaps in the literature:

1. The study will determine the effectiveness of peer navigation services for biomedical HIV prevention; SUD treatment; and HIV, STI, and HCV screening among MSM and transgender women with SUDs leaving incarceration.
2. The study will examine the acceptability and feasibility of combining peer mentor services with a mobile app to facilitate service utilization and participant-peer communication. To our knowledge, no other study combines these interventions.
3. The study will assess patterns of PrEP uptake and utilization in MSM and transgender women leaving jail, data that are critical to local jurisdictions’ abilities to reach goals for the reduction of new HIV infections.
4. Finally, the study will provide heretofore unavailable data on postincarcerated persons’ HIV and STI screening, PrEP use, substance use treatment, and service utilization patterns and experiences during re-entry, including geocoded data for those in the two intervention arms. These data may inform resource allocation and strategic planning by policy makers, planners, and other stakeholders.

Peer navigation and peer support have been shown to be acceptable, effective, and cost-efficient strategies for reaching HIV-positive and -negative MSM and transgender women to promote HIV prevention, HIV testing, linkage to HIV primary care, retention in primary care, and viral suppression [32]. However, the efficacy of their use with HIV-negative people leaving jail has not been established. A recent study by Nyamathi et al that compared varying levels of peer coach and nurse-partnered interventions on re-arrests showed no differences from usual care in rates of re-arrest among men on parole who were recruited from in-patient substance abuse treatment centers [33]. However, their nurse case-management model has shown evidence of effectiveness for improvements in hepatitis A and B vaccine uptake and decreased substance use [27,34]. Expanding the evidence base of effective peer-based approaches is particularly critical now that California and other states are expanding Medicaid reimbursements to cover peer and patient navigation, creating equitable reimbursement rates for substance abuse treatment, and expanding treatment programs to within jails and prisons [35].

The Linking Inmates to Care in Los Angeles (LINK LA) study enrolled 356 HIV-positive people from an MSM and transgender women’s jail unit into an RCT comparing a manualized peer mentor intervention to standard transitional case management (TCM). Adjusted probabilities of viral suppression remained stable in the intervention group, from 0.488 at baseline to 0.485 at 12 months; in the TCM group, it declined from 0.520 at baseline to 0.300 at 12 months (*P*=.002). The Passport to Wellness study has enrolled 92 eligible African American MSM to date in an RCT comparing those assigned to receive a passport, or personalized set of referrals with accompanying incentives for utilization, and the support of a trained peer mentor to those only receiving the passport and incentives. Both groups saw substantial increases in HIV testing, and new enrollment in PrEP increased more for the intervention group than the control group. However, data are very preliminary as, to date, follow-up has not been completed. The proposed study will incorporate elements of both the LINK LA and Passport to Wellness interventions and will enhance them further with the development of GeoPass, which is designed to enhance peer-based interventions and facilitate the use of services along the HIV prevention continuum.

Mobile technology use has become ubiquitous. It is something that individuals of all ages, sexualities, gender statuses, socioeconomic positions, and races and ethnicities regularly utilize in multiple aspects of their daily lives, because it facilitates and enables interactions with information, individuals, businesses, and providers. Service providers can move *one step ahead* by finding efficient and feasible ways to engage these technologies with their participants. To reach marginalized and stigmatized populations who bear a disproportionate share of risk for HIV and other STIs, new initiatives to address HIV prevention must efficiently merge approaches that marshal existing and emerging mobile technologies with those involving more intensive, direct human contact. By enabling researchers and providers the ability to track and provide feedback on participants’ service utilization, and by facilitating real-time interaction between participants and peer mentors, web-based mobile technology has the potential to increase utilization and contribute to improved care delivery along the HIV prevention and substance use treatment continua.

## Appendix

Multimedia Appendix 1 Sample Mobile-Enhanced Prevention Support (MEPS) wellness passport.

## Abbreviations

ASAM: American Society of Addiction Medicine
CHIPTS: Center for HIV Identification, Prevention, and Treatment Services
CHRP: California HIV/AIDS Research Program
CTSI: Center for Translational Sciences Institute
DSM-5: Diagnostic and Statistical Manual of Mental Disorders, Fifth Edition
GLMM: generalized linear mixed model
HCV: hepatitis C virus
K6G: Keep Away Designation 6G
LAC: Los Angeles County
LA CADA: Los Angeles Centers for Alcohol and Drug Abuse
LASD: Los Angeles County Sheriff's Department
LINK LA: Linking Inmates to Care in Los Angeles
Medi-Cal: Medicaid in California
MEPS: Mobile-Enhanced Prevention Support
MSM: men who have sex with men
NIMH: National Institute of Mental Health
NIMHD: National Institute on Minority Health and Health Disparities
PrEP: pre-exposure prophylaxis
RCT: randomized controlled trial
SCT: Social Cognitive Theory
START: Substance Treatment And Re-entry Transition
STI: sexually transmitted infection
SUD: substance use disorder
TCM: transitional case management
UCLA: University of California, Los Angeles
