# Supplementary figures and images for: Mobile-Enhanced Prevention Support Study for Men Who Have Sex With Men and Transgender Women Leaving Jail: Protocol for a Randomized Controlled Trial
Source: JMIR Res Protoc. 2020 Sep 22;9(9):e18106. doi: 10.2196/18106 (PMC7539160; doi:10.2196/18106)

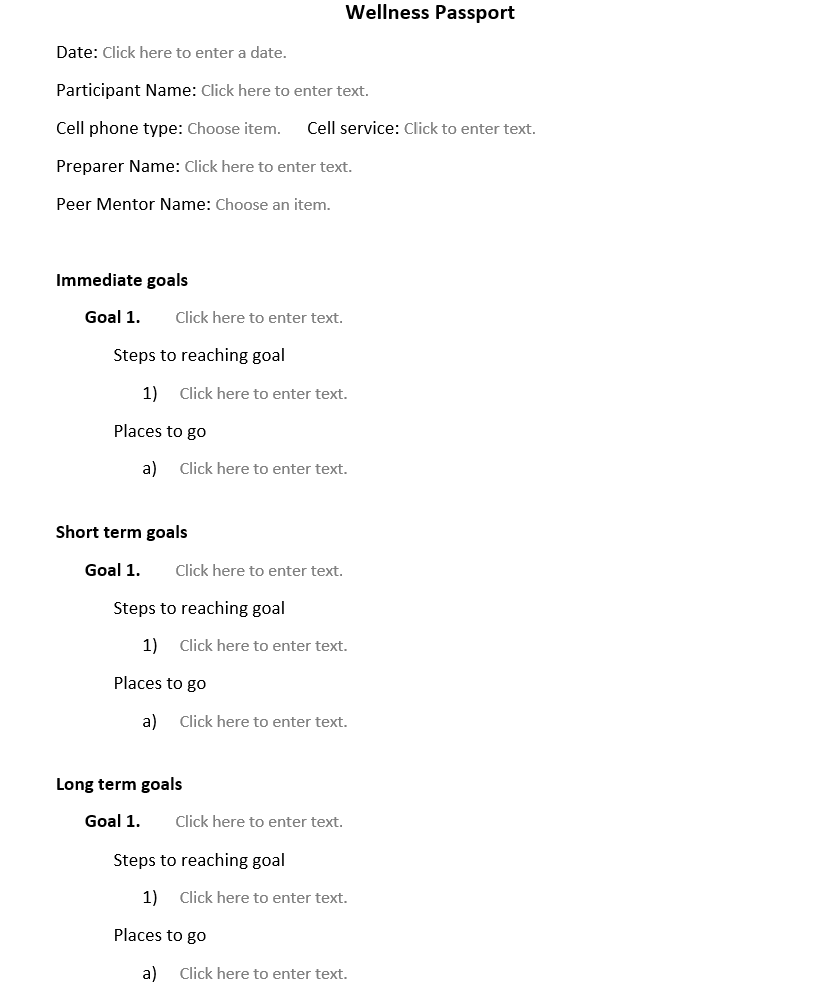

Supplement: Multimedia Appendix 1 [file resprot_v9i9e18106_app1.png]
